# Production of β-methylamino-L-alanine (BMAA) and Its Isomers by Freshwater Diatoms

**DOI:** 10.3390/toxins11090512

**Published:** 2019-09-02

**Authors:** Jake P. Violi, Jordan A. Facey, Simon M. Mitrovic, Anne Colville, Kenneth J. Rodgers

**Affiliations:** 1 Neurotoxin Research Group, School of Life Sciences, Faculty of Science, The University of Technology Sydney, Ultimo 2007, Australia; 2 Freshwater and Estuarine Research Group, School of Life Sciences, Faculty of Science, The University of Technology Sydney, Ultimo 2007, Australia

**Keywords:** BMAA, 2,4-DAB, AEG, diatom, Bacillariophyceae, algal toxins, non-protein amino acids, amino acids

## Abstract

β-methylamino-L-alanine (BMAA) is a non-protein amino acid that has been implicated as a risk factor for motor neurone disease (MND). BMAA is produced by a wide range of cyanobacteria globally and by a small number of marine diatoms. BMAA is commonly found with two of its constitutional isomers: 2,4-diaminobutyric acid (2,4-DAB), and N-(2-aminoethyl)glycine (AEG). The isomer 2,4-DAB, like BMAA, has neurotoxic properties. While many studies have shown BMAA production by cyanobacteria, few studies have looked at other algal groups. Several studies have shown BMAA production by marine diatoms; however, there are no studies examining freshwater diatoms. This study aimed to determine if some freshwater diatoms produced BMAA, and which diatom taxa are capable of BMAA, 2,4-DAB and AEG production. Five axenic diatom cultures were established from river and lake sites across eastern Australia. Cultures were harvested during the stationary growth phase and intracellular amino acids were extracted. Using liquid chromatography triple quadrupole mass spectrometry (LC-MS/MS), diatom extracts were analysed for the presence of both free and protein-associated BMAA, 2,4-DAB and AEG. Of the five diatom cultures analysed, four were found to have detectable BMAA and AEG, while 2,4-DAB was found in all cultures. These results show that BMAA production by diatoms is not confined to marine genera and that the prevalence of these non-protein amino acids in Australian freshwater environments cannot be solely attributed to cyanobacteria.

## 1. Introduction

### 1.1. Algal and Diatom Toxins

Many algal groups, including cyanobacteria, dinoflagellates and diatoms, are capable of forming dense blooms under favourable conditions. Algal blooms are of great epidemiological and ecological significance since they produce a diverse range of chemicals which can cause toxic effects to humans and livestock, including skin irritation (dermatoxins), hepatic damage (hepatotoxins), and neuronal damage (neurotoxins) [1,2]. The production of toxins by diatoms was first reported in the 1980s, when the marine and estuarine diatom genus *Pseudo-nitzschia* was found to produce the neurotoxic amino acid domoic acid (DA) [3]. Until recently, DA was thought to be the only toxin produced by diatoms, however three reports have now been published on the production of β-methylamino-L-alanine (BMAA) and its isomer 2,4-diaminobutyric acid (2,4-DAB) by marine diatoms [4,5,6]. DA, BMAA and 2,4-DAB have only been detected in marine diatoms and it is unknown whether freshwater diatoms produce BMAA or 2,4-DAB.

### 1.2. The Toxicity of BMAA, 2,4-DAB and AEG

BMAA and its two constitutional isomers 2,4-DAB and N-(2-aminoethyl)glycine (AEG) can all be categorised as non-protein amino acids (NPAA), meaning that they are amino acids not encoded in ribosomal protein synthesis [7]. AEG is the least toxic of the three isomers; studies in a human neuroblastoma cell line (SH-SY5Y) found it to be four times less toxic than 2,4-DAB and BMAA [8], and it was also found to be 10,000 times less toxic than BMAA in an *Artemia salina* bioassay [9]. BMAA and 2,4-DAB have been shown to be excitotoxic via the formation of β-carbamate adducts in the presence of bicarbonate. These β-carbamate adducts are capable of activating glutamate receptors [10,11]. While this acute toxicity has been identified in vitro, the time lag between human exposure to BMAA and the emergence of clinical symptoms suggests that BMAA also has a chronic mechanism of toxicity [12]. This chronic toxicity has been hypothesised to result from the ability of BMAA to cause damage to proteins or cause protein misfolding [13,14,15]. BMAA is generally found in both free and protein-associated forms [16]. The nature of this association with protein is not clear [17,18] but one hypothesis is that BMAA is mistakenly incorporated into the polypeptide chain of proteins in place of L-serine [14] or other protein amino acids [19] in a process known as protein amino acid mimicry [20]. The misfolding of proteins in vivo following BMAA exposure could result in the deposition of protein aggregates in neurons, as occurs in BMAA-treated primates [13,15]. The link between BMAA exposure and neurodegeneration has been questioned on a number of levels [21]. For example, the BMAA-serine exchange was proposed based primarily on in vitro studies using radiolabelled BMAA [14] and this has not been independently confirmed using chemical analyses [22]. An overview of 50 years of BMAA research by Peter Nunn, a member of the team that discovered and first synthesized BMAA, provides an excellent summary of the field and outlines the complex issues that have still to be unraveled on the possible involvement of BMAA in Guamanian ALS-PDC and in neurological diseases today [23].

### 1.3. BMAA and Its Worldwide Distribution

BMAA was first linked to neurodegenerative diseases on the island of Guam in the 1950s, where a high incidence of a disease complex known as amyotrophic lateral sclerosis-Parkinsonism-dementia complex (ALS-PDC) was reported. It is unclear how close Guamanian ALS-PDC is to what is currently classified as ALS, Parkinsonism or dementia [21]. The Chamorro people had a 50–100 times higher incidence of these neurodegenerative diseases than the global average [24,25,26]. The disorder was linked to lifestyle rather than the genetic background of the island’s inhabitants since the incidence dropped rapidly with the adoption of American habits and diet, providing support to the theory that environmental factors were involved [27,28]. BMAA originated from cyanobacteria living in the roots of the cycad palms and it biomagnified through the Guamanian food web, resulting in exposure and consumption by the islanders [29,30,31]. BMAA has now been detected not only in the terrestrial environmental but in marine and freshwaters globally. BMAA has also been shown to bioaccumulate through freshwater food webs in Sweden [32] and marine food webs from several locations, causing it to being present in seafood [33,34,35]. A correlation between increased incidences of ALS and living close to water bodies has been established, with these water bodies being known to have frequent algal blooms which may contain BMAA [36,37,38,39]. Inhalation of aerosolised BMAA has been identified as a potential route of exposure and may help explain the increased occurrence of neurodegenerative hotspots around lakes with regular algal blooms [36,40]. Accurate quantification of BMAA in complex matrices is a contentious issue and has been comprehensively reviewed elsewhere [41,42]. In the present study, propyl chloroformate derivatisation of amino acids was employed, followed by liquid chromatographic separation and mass spectrometric detection, a validated method that has been described in two previous studies [39,43].

### 1.4. Producers of BMAA, 2,4-DAB & AEG

Cox et al. (2005) showed that both symbiotic and free-living cyanobacteria from a variety of locations have the capacity to produce BMAA [44]. In that study, quantification came under question due to its reliance on fluorescence detection [45], however, later studies confirmed these results via the use of tandem mass spectrometry detection methodologies [5,39,43,46]. More recent studies looking at BMAA alongside its isomers determined that a large array of cyanobacteria can also produce 2,4-DAB and AEG [4,39,47,48]. Despite the large number of studies investigating BMAA, 2,4-DAB and AEG synthesis by cyanobacteria, there are relatively few studies investigating their synthesis by diatoms. At the time of writing, there were only three studies reporting BMAA, 2,4-DAB and AEG production by diatoms [4,5,6], with another three focusing on production kinetics [49,50,51]. All these studies focused on marine diatom species and none investigated freshwater diatoms or diatoms originating from Australia.

### 1.5. Aims of the Study

The aim of the present study was to isolate several genera of freshwater diatoms from eastern Australian waterways and use liquid chromatography triple quadrupole mass spectrometry to determine if Australian freshwater diatoms are capable of BMAA, 2,4-DAB and AEG production.

## 2. Results

A total of five diatom cultures were established, one from each site sampled, with five different genera being identified via morphology. BMAA and AEG were detected in four of the five cultures (Table 1 & Table 2). *Fragilaria* did not have detectible levels of BMAA, and *Cyclotella* did not have detectable levels of AEG. 2,4-DAB was detected in all the cultures (Table 3). For all amino acid concentrations, both per cell and per dry weight, the concentration in the bound fraction was always higher than the free fraction counterpart.

BMAA was detected in the free fraction for two of the five diatom cultures, *Cyclotella* and *Navicula,* and four of the five bound fractions, being absent from the *Fragilaria* culture, which had no detectable BMAA from either fraction. 2,4-DAB was detected in all free fractions and three out of five bound fractions and was absent from both the *Fragilaria* and *Aulacoseira* bound fractions. AEG was detected in four out of five free fractions and bound fractions, with the *Cyclotella* culture having no detectable AEG in either fraction.

The highest BMAA, 2,4-DAB and AEG concentrations were detected in the bound fraction of the *Navicula* culture at 369.64 ng/g, 4678.75 ng/g and 1328.11 ng/g per dry weight (DW) respectively, or 4.45 fg/cell, 56.37 fg/cell and 16 fg/cell per cell. The lowest BMAA concentration per dry weight and per cell was detected in the bound fraction of the *Tabellaria* culture at 19.99 ng/g and 0.34 fg/cell. The lowest 2,4-DAB concentration per dry weight and per cell was detected in the free fraction of the *Aulacoseira* culture at 103 ng/g and 2.3 fg/cell. The lowest AEG concentration per dry weight and per cell was detected in the free fraction of the *Aulacoseira* culture at 49.55 ng/g and 1.1 fg/cell.

## 3. Discussion

### 3.1. Comparison of Diatom Studies

The current study is the first to show BMAA production in freshwater diatoms. Five genera isolated from five locations across eastern Australia were examined for BMAA, 2,4-DAB and AEG. BMAA and AEG were detected in four out of the five cultures and 2,4-DAB was detected in all the cultures. Three previous studies detailed diatom production of BMAA in marine species. The first study to show the production of BMAA from marine diatoms was by Jiang et al. (2014) [6]. In the study, six marine diatoms species were examined and all had detectable levels of BMAA. While some species were stated to have detectable levels of 2,4-DAB and AEG, the specific species were not reported. Réveillon et al. (2015) [4] detected BMAA in four out of eight marine diatom cultures, AEG in one culture and 2,4-DAB in all cultures. The most recent study by Lage et al. (2016) [5] reported on nine marine diatom cultures producing BMAA with no testing of 2,4-DAB or AEG. The results from the present study support a hypothesis first suggested by Jiang et al. (2014) [6] that BMAA production may be a common trait amongst all diatoms. 2,4-DAB production may be even more common than BMAA, since 2,4-DAB was detected in all cultures in this study as well as in the study by Réveillon et al. (2015) [4].

### 3.2. BMAA in Freshwater Diatoms

The present study examined five genera of freshwater diatoms for BMAA and its isomers: *Aulacoseira, Cyclotella, Fragilaria, Navicula and Tabellaria.* Of the five examined, only members of the genus *Navicula* had been previously examined for BMAA production in marine taxa. Both Jiang et al. (2014) [6] and Lage et al. (2016) [5] examined the marine species *Navicula pelliculosa* (CCAP 1095/1 and 1095/9) and found detectible levels of BMAA, however, both studies lacked information regarding 2,4-DAB and AEG, with quantification of BMAA in *Navicula pelliculosa* limited to one study by Lage et al. (2016) [5]. Lage et al. (2016) [5] found higher BMAA concentrations in the bound fraction of *Navicula* compared to free, which is consistent with the findings of this study. The free fraction was higher in the present study (151 ng/g) compared to Lage et al. (2016) [5] (91 ng/g), while the bound fraction was lower (369 ng/g vs. 1768 ng/g). This disparity is most likely due to several factors, including differences among species in a genus, growth conditions and origin of habitat [50,52].

The remaining three genera that had detectable BMAA—*Aulacoseira, Cyclotella and Tabellaria*—have not been tested in other studies. The only genus with no detectable BMAA was *Fragilaria*, which also has yet to be tested in other BMAA studies. Overall, the concentrations of BMAA were generally lower than those of the marine diatom studies, however, there is a lack of knowledge regarding free vs. bound concentrations of BMAA in diatoms, as only Lage et al. (2016) [5] separately analysed both bound and free BMAA. The other two studies pooled the two fractions together for a total BMAA measurement [4,6].

When compared to BMAA production by cyanobacteria, production by diatoms appears to be notably less. For example, in a similar study, Violi et al. (2018) [39] found that some cyanobacterial cell quotas were an order of magnitude higher than those found in the present study. The highest concentrations of BMAA production in dry weight by diatoms was comparable to the lowest by cyanobacteria [39]. However, a comparison to cyanobacterial BMAA concentrations in dry weight is tenuous due to the increased mass of the diatoms frustule [6]. Cell quota can be used as a metric for comparison, however, few cyanobacterial BMAA studies calculate this and most instead use dry weight measurements [43,44,53,54,55,56]. Those that used cell quota show that cell quota for diatoms is similar to the lower concentrations observed in cyanobacteria [39]. BMAA production can be influenced by environmental factors. For example, nitrogen starvation can promote BMAA and 2,4-DAB production in diatoms [50] and BMAA in cyanobacteria [57]. Therefore, culturing conditions may be an important factor influencing the detected concentrations of BMAA, 2,4-DAB and AEG in the present study.

### 3.3. AEG and 2,4-DAB in Freshwater Diatoms

Diatom production of 2,4-DAB and AEG is severely understudied, with only Réveillon et al. (2015) [4] reporting total 2,4-DAB and AEG levels, and Jiang et al. (2013) [6] conducting a brief qualitative study. This is despite the neurotoxic properties of 2,4-DAB, the typically high concentrations and commonality in both environmental samples and cultures [4,39,43,58]. Indeed, 2,4-DAB was the most consistently detected of the BMAA isomers, being present in all cultures, and was found at higher concentrations than BMAA. This is consistent with Réveillon et al. (2015) [4], who observed 2,4-DAB in more diatom cultures than BMAA and at higher concentrations. The higher occurrence of 2,4-DAB in environmental samples compared to BMAA may be explained by the production of 2,4-DAB by other organisms beside cyanobacteria and diatoms [59,60]. It is unknown why diatoms would have higher levels of 2,4-DAB than BMAA, although this may be due to an underlying metabolic function of 2,4-DAB. As with BMAA, 2,4-DAB levels were typically lower (both per dry weight and cell quota) compared to cyanobacteria [39,43]. For all isomers, the bound fraction was found to have the highest concentrations, however, as the nature of the bound fraction in both cyanobacteria and diatoms is still unknown, it remains to be determined whether this is of any biological importance.

Like BMAA, AEG was detected in all but one culture; only *Cyclotella* had no detectable levels of AEG. This suggests that like BMAA and 2,4-DAB, AEG may also be commonly produced by freshwater diatoms. While not known to be toxic to humans, it is important to know that AEG can be produced by diatoms so that when examining cultures or environmental samples, it can be ensured that there is no misidentification with its toxic isomers. The only diatom species previously examined for AEG, *Skeletonema pseudonana*, had total AEG levels that were lower than the BMAA levels in that study [4]. In the present study, AEG levels were higher than BMAA in both fractions, which is a similar trend to Australian cyanobacterial isolates [39].

### 3.4. Toxic Diatoms in Freshwater Systems

BMAA and its isomers have previously been detected in cyanobacterial blooms and isolates from eastern Australia [39,43]. This study demonstrates that diatoms may be an additional source of BMAA and 2,4-DAB in freshwater environments in Australia and potentially around the world. This may represent a health risk to people living around freshwater sources due to potential BMAA exposure via drinking water contamination, aerosolisation and/or the consumption of food sourced from freshwater systems undergoing diatom blooms or food web bioaccumulation. Cyanobacteria are the primary concern when managing harmful algal blooms in freshwater systems as there has previously been little evidence of toxin production by other algal groups. Given the abundance of these diatoms in freshwater systems, their position at the foundation of the foodweb and propensity for BMAA to biomagnify [32,33,34,35], diatom blooms may need to be considered in water quality programs and monitored alongside cyanobacteria to ensure safe drinking, recreational and irrigation waters. While several genera in this study regularly form dense blooms [61,62,63], further research is required to determine what concentrations of BMAA and 2,4-DAB are present in freshwater diatom blooms. The geographic extent of BMAA-containing blooms and how environmental factors influence toxin production in freshwater diatoms are other potential areas of further research.

## 4. Conclusions

This study examined whether the neurotoxin BMAA and its isomers 2,4-DAB and AEG were produced by freshwater diatoms isolated from several locations across eastern Australia. Five axenic cultures were established and four were found to have detectable levels of BMAA and AEG, while all were found to have detectable levels of 2,4-DAB. These results are the first report of freshwater diatoms producing BMAA and 2,4-DAB. It also suggests that the presence and abundance of BMAA in freshwater ecosystems is not solely attributed to cyanobacteria but may also be from diatoms.

## 5. Materials and Methods

### 5.1. Sample Collection

**Figure 1:**
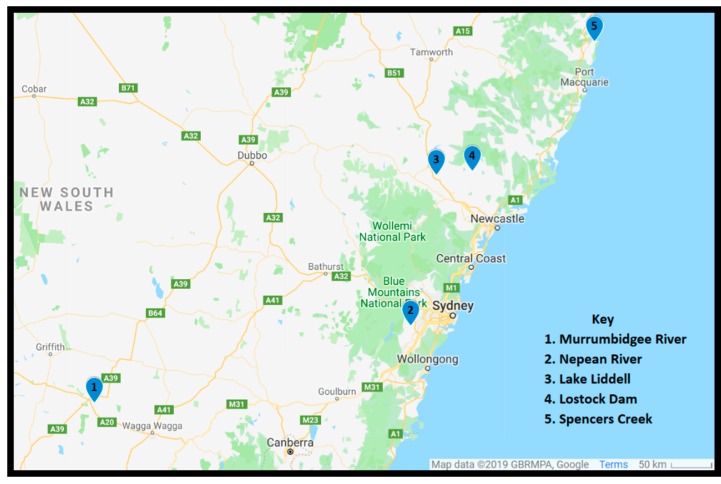
Map of sites sampled.

A total of five sites were sampled and were selected to include a diverse array of freshwater locations in eastern Australia (Figure 1). Sampling was conducted between March 2017 and July 2018. Phytoplankton was concentrated using a 20-μm plankton net and poured into a 200-mL polyethylene terephthalate (PET) bottle. Once returned to the laboratory, samples were kept at ambient temperature.

### 5.2. Diatom Isolation, Purification & Culturing

#### 5.2.1. Algal Media

Two freshwater growth media, MLA and BG-11 with addition of 1 g/L sodium metasilicate (Na_2_SiO_3_) (Sigma-Aldrich, Castle Hill, NSW, Sydney, Australia), added to supply a source of silica for the diatoms, were used to isolate freshwater diatoms. Stock solutions of MLA medium were purchased from AusAqua Pty. Ltd. Wallaroo, South Australia (AlgaBoostTM four-part concentrate) [64]. Stocks of BG-11 were modified from the University of Texas (UTEX) recipe [65,66]. MLA and BG-11 media were prepared from their respective stocks and sterilised in an autoclave (Tangent Tiger Sterilizer chamber, Atherton, Victoria, Australia). For each site sampled, two Falcon 24-well multi-well plates were set up and 1.5-mL culture medium was added to each well, MLA for one plate and BG-11 for the other.

#### 5.2.2. Diatom Isolation

Concentrated algal samples were transferred to a microscope slide to determine if any diatom species were present. After identifying diatom species, 50 μL of sample was transferred to a new microscope slide and diluted with 200 μL of the corresponding medium in the well plate that was being used. Using an elongated fine-tipped Pasteur pipette, desired diatom species were transferred to another slide, then further diluted with the selected medium. This was repeated until only the desired diatom species was visible under the microscope, and it was then transferred to a well of the corresponding well plate. Well plates were left in an incubator (Labec, ICBOD140 incubator, Labec, Marrickville, NSW Australia) set to 24 °C with light conditions of 17–20 μmol/m^2^/s, 14–10 h light to dark cycle for two weeks, to allow the isolates to grow. After two weeks, individual wells were assessed via light microscopy on their level of contamination. Isolation was repeated into new well plates if deemed necessary to ensure that any contamination was eliminated to produce a mono-species culture.

#### 5.2.3. Diatom Purification and Culturing

Once deemed to be mono-species, diatom isolates were cultured in 250-mL conical flasks containing 110 mL of the desired medium. Isolates were transferred into a conical flask with sterile medium under a sterile environment using a biosafety cabinet (Purifier Logic+ Class II, Type A2 Biosafety Cabinets, Labconco, Kansas, USA). Cultures were grown in an environmental chamber (Labec, HC-50 environmental chamber, Labec, Marrickville, NSW Australia) set to 24 °C under 20–25 μmol/m^2^/s photons with a 14–10 h light-to-dark cycle. To ensure that cultures were axenic, all cultures were treated with a mixture of antibiotics (Tetracycline, Penicillin G, Neomcyin and Chloramphenicol at 100 μg/mL) (Sigma-Aldrich, Castle Hill, NSW, Sydney, Australia) during subculturing as it has been suggested that 2,4-DAB found in some diatom cultures may be due to the presence of bacteria [51]. Cell counts to determine stage of growth cycle were carried out by loading 1 mL of culture into a Sedgwick-Rafter cell counting chamber and viewing with a compound microscope (Olympus BX41, Olympus Australia Pty Ltd, Notting Hill, VIC Australia).

### 5.3. Amino Acid Extraction

#### 5.3.1. Diatom Harvesting, Lysing and Fractionation

Diatom cultures (100 mL) were harvested during the stationary phase, then subjected to centrifugation (10 min at 3500× *g*). Then, the supernatant was decanted and the remaining pellet was stored at −80 °C. The samples were freeze-dried (Martin Christ, alpha 2-4 LD plus) at 0.1 mbar and −80 °C for 24 h to ensure sublimation of any liquid remaining. The freeze-dried pellet was weighed and then submerged in 300 μL 10% *w*/*v* trichloroacetic acid (TCA) (TCA ≥ 99.5%, Sigma-Aldrich, Castle Hill, NSW, Sydney, Australia) to precipitate the protein. The sample then underwent probe sonication (Vibra Cell, VC50T 50 watt Ultrasonic Processor, Sonics & Materials, Connecticut, USA) for 1.5 min at 70% power. To ensure complete lysis of cells, this was carried out in duplicate with samples being left on ice for 1 min between repeats. Samples were left to precipitate overnight at 4 °C. Samples were then centrifuged at 3500× *g* for 15 min at 8 °C, with the supernatant being transferred to a new 2-mL tube deemed the “free fraction”. The remaining pellet was then extracted twice by adding 300 μL of 10% TCA, resuspending and centrifuging again at 3500× *g* for 15 min at 8 °C, with the supernatant being transferred to the free fraction tube. The pellet was then reconstituted in 300 μL of 10% TCA and centrifuged once again at 3500× *g* for 15 min at 8 °C, and the supernatants were transferred to the free fraction. The remaining pellet was transferred to a shell vial with two washes of 10% TCA in acetone (100 μL). The shell vial was centrifuged (Eppendorf™, MiniSpinPlus™ Microcentrifuges) at 5300× *g* for 5 min, with the supernatant being transferred to the free fraction. The free fraction was then evaporated using a centrifugal evaporator (Thermo Fisher Scientific, Savant DNA 120 Speedvac concentrator) for 24 h to remove volatile liquids, then frozen and freeze-dried at 0.1 mbar to remove any remaining liquid. The free fraction was reconstituted in 200 μL of 20 mM hydrochloric acid (HCl) (HCl, 37%, Sigma-Aldrich, Castle Hill, NSW, Sydney, Australia) and was then stored at −80 °C.

#### 5.3.2. Protein Hydrolysis

The shell vial containing the final pellet from the extraction of the free fraction (Section 5.3.1) was centrifuged in the centrifugal evaporator to remove any remaining TCA acetone from the pellet. An amount of 1 mL of 6 M HCl was transferred to a vacuum hydrolysis vial and the shell vial was placed inside it. To avoid oxidation of the proteins and ensure that protein was hydrolysed correctly, oxygen was removed from the hydrolysis vial under vacuum (300 mbar) and then replaced with nitrogen gas. This was carried out in triplicate to ensure that as little oxygen remained in the vial as possible. The vacuum vials were incubated for 16 h in an oven set at 110 °C. After 16 h, the vacuum vials were removed and left to cool after having the pressure released for 10 min. The shell vials were removed from the vacuum vials and the hydrolysed pellet was reconstituted in 200 μL of 20 mM HCl. The shell vials were then centrifuged for 2 min at 5300× *g*. The supernatant was transferred to a new 2-mL tube deemed the “protein fraction” and store at −80 °C. Both the free and protein fraction samples were transferred to a 0.2 membrane filter (Ultrafree-MC LG Centrifugal 0.2 μm pore size PTFE Membrane Filter (UFC30LG25)) in a 2-mL tube and centrifuged for 30 min at 5000× *g*. The remaining amino acid extracts from both fractions were stored at −80 °C.

### 5.4. Amino Acid Derivatisation and LC-MS/MS

#### 5.4.1. Propyl Chloroformate Derivatisation

An amount of 100 μL of amino acid extract was diluted 1:2 with 1 ng/μL of the internal standard deuterated-DAB (D5-DAB) (CDN Isotopes, Pointe-Claire, Quebec, Canada) to account for loss during derivatisation or instrumentation issues. Amino acid extracts with the internal standard underwent propyl chloroformate derivatisation using the Phenomenex^®^ EZ:Faast™ amino acid analysis kit (Phenomenex^®^ Australia, Lane Cove, NSW, Australia) as per instructions from the manufacturer. After derivatisation, derivatised amino acids were reconstituted in 50 μL of the starting chromatography mobile phase, 45% ultrapure water (Solvent A) and 55% Methanol (Solvent B) (HPLC-grade, Honeywell Burdick & Jackson, Muskegon, MI, USA), both buffered with 0.1% (*v*/*v*) formic acid (Sigma Aldrich, Castle Hill, NSW, Australia). The derivatised amino acids were then transferred to an autosampler vial. For each LC-MS/MS run, a 6-point calibration curve (250 pg/μL 100 pg/μL, 50 pg/μL 25 pg/μL 10 pg/μL, 1pg/μL) was run alongside samples for accurate quantification. Standards of each analyte (BMAA (Sigma-Aldrich, Castle Hill, NSW, Australia), 2,4-DAB and AEG (both, Toronto Research Chemicals Inc. North York, ON, Canada) were used to construct the calibration curve.

#### 5.4.2. LC-MS/MS Analysis

**Figure 2:**
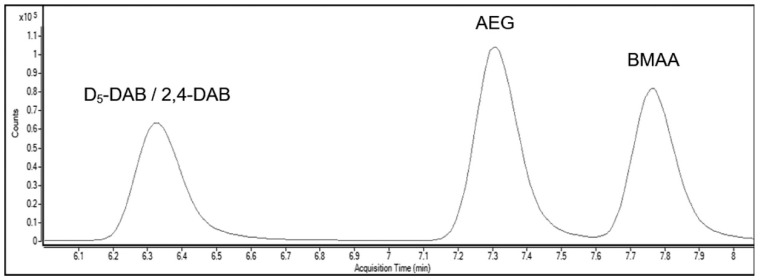
Liquid chromatograph of standards containing all isomers, used in the calibration curve (Violi et al., 2019) [39].

The LC-MS/MS method used was developed by Main et al. (2018) [43] and was carried out on an Agilent 1290 infinity LC system and a Agilent 6490 triple quadrupole LC-MS. LC was carried out on a Kintex^®^ C18 column (17 μm particle size, 100 Å pore size) (Phenomenex^®^ Australia, Lane Cove, NSW, Australia), via a gradient elution of the mobile phases (Solvent B’s gradient was as follows: 0.00 min 55%, 10.00 min 68%, 10.10 min 100%, 15.00 min 100%, 15.10 min 55%) with a flow rate of 0.250 mL/min with a column temperature of 35 °C (a typical chromatogram is shown in Figure 2). After each sample, the mobile phase was left at starting conditions for 2 min for column re-equilibration. Samples were run in triplicate injections of 5 μL. Prior to analysis, multiple reaction monitoring (MRM) ion transitions were set up for each analyte and the internal standard (D5-DAB) (Table 4). Data acquisition occurred in electrospray ionisation (ESI) positive mode, the drying gas temperature was 250 °C at 14 L/min, the sheath gas temperature was 250 °C at 11 L/min, with a nebuliser pressure of 20 psi. The limit of detection and the limit of quantification were determined with a signal to noise ratio of 3.3 and 10 respectively. BMAA was calculated to have a LOD of 0.02 pg/μL and a LOQ of 0.05 pg/μL, while both AEG and 2,4-DAB were calculated to have a LOD of 0.04 pg/μL and a LOQ of 0.13 pg/μL.

## Figures and Tables

**Table 1:** β-methylamino-L-alanine (BMAA) concentrations ± standard error of the mean (*n* = 3) in diatom cultures (Maximum value per DW = 369.64 ng/g, per cell = 4.45 fg/cell). ND denotes not detected.

Genera	Location	Free BMAA (ng/g DW)	Bound BMAA (ng/g DW)	Free BMAA Per Cell (fg/Cell)	Bound BMAA Per Cell (fg/Cell)
*Aulacoseira*	Nepean River	ND	40.28 ± 0.83	ND	0.89 ± 0.02
*Cyclotella*	Lake Liddell	103.58 ± 1.94	154.06 ± 4.94	2.69 ± 0.05	4.01 ± 0.12
*Fragilaria*	Murrumbidgee River	ND	ND	ND	ND
*Navicula*	Lostock Dam	151.2 ± 10.02	369.64 ± 11.96	1.82 ± 0.12	4.45 ± 0.14
*Tabellaria*	Spencers Creek	ND	19.99 ± 1.39	ND	0.34 ± 0.02

**Table 2:** N-(2-aminoethyl)glycine (AEG) concentrations ± standard error of the mean (*n* = 3) in diatom cultures (Maximum value per DW = 1328.11 ng/g, per cell = 13.09 fg/cell). ND denotes not detected.

Genera	Location	Free AEG (ng/g DW)	Bound AEG (ng/g DW)	Free AEG Per Cell (fg/Cell)	Bound AE Per Cell (fg/Cell)
*Aulacoseira*	Nepean River	49.55 ± 3.29	196.17 ± 4.93	1.1 ± 0.07	4.38 ± 0.11
*Cyclotella*	Lake Liddell	ND	ND	ND	ND
*Fragilaria*	Murrumbidgee River	323.05 ± 28.75	1162.12 ± 43.97	3.64 ± 0.32	13.09 ± 0.49
*Navicula*	Lostock Dam	563.5 ± 1.57	1328.11 ± 25.31	6.78 ± 0.02	16 ± 0.30
*Tabellaria*	Spencers Creek	124.83 ± 0.7	150.19 ± 1.12	2.15 ± 0.01	2.59 ± 0.02

**Table 3:** 2,4-DAB concentrations ± standard error of the mean (*n* = 3) in diatom cultures (Maximum value per DW = 4678.75 ng/g, per cell = 56.37 fg/cell). ND denotes not detected.

Genera	Location	Free 2,4-DAB (ng/g DW)	Bound 2,4-DAB (ng/g DW)	Free 2,4-DAB Per Cell (fg/Cell)	Bound 2,4-DAB Per Cell (fg/Cell)
*Aulacoseira*	Nepean River	103.27 ± 9.96	ND	2.3 ± 0.22	ND
*Cyclotella*	Lake Liddell	113.7 ± 2.13	169.12 ± 5.43	2.96 ± 0.05	4.4 ± 0.14
*Fragilaria*	Murrumbidgee River	258.57 ± 24.06	ND	2.91 ± 0.27	ND
*Navicula*	Lostock Dam	594.81 ± 53.22	4678.75 ± 83.2	7.16 ± 0.64	56.37 ± 1.0
*Tabellaria*	Spencers Creek	146.16 ± 8.8	248.48 ± 17.43	2.52 ± 0.15	4.28 ± 0.30

**Table 4:** MRM ion transitions for all target amino acids. * denotes peaks used for quantification (Violi et al., 2019) [39].

Amino Acid	Retention Time (min)	Precursor Ion (*m*/*z*)	Product Ion (*m*/*z*)
BMAA	7.80	333	73 99.1 159.1 187.1 *
2,4-DAB	6.30	333	56 99 187.1 273.1 *
AEG	7.30	333	56.1 88* 99 187.1
D5-DAB	6.30	339	102.9 103.9 192 278.1 *
